# Recurrent Pulmonary Nodules, from Pulmonary Cryptococcosis to ANCA-Associated Vasculitis: A Case Report

**DOI:** 10.2174/0115734056454815260316084630

**Published:** 2026-03-30

**Authors:** Xingxing Zhu, Xiaohong Wu, Yahong Sun, Shengjie Wu, Jilin Zeng, Zhihao Liu, Jing Zheng, Qifan He

**Affiliations:** 1 Department of Pulmonary and Critical Care Medicine, Affiliated Haining Hospital of Jiaxing University, Haining People's Hospital, Haining, China; 2 Department of Pulmonary and Critical Care Medicine, Sir Run Run Shaw Hospital, Zhejiang University School of Medicine, Hangzhou, China; 3 Department of Pharmacy, Sir Run Run Shaw Hospital, Zhejiang University School of Medicine, Hangzhou, China; 4 Department of Respiratory Disease, Thoracic Disease Center, The First Affiliated Hospital, College of Medicine, Zhejiang University, Hangzhou, China; 5 Department of Radiology, Affiliated Haining Hospital of Jiaxing University, Haining People's Hospital, Jiaxing, China

**Keywords:** Pulmonary nodule, Pulmonary cryptococcosis, Microscopic polyangiitis, Antineutrophil cytoplasmic antibody-associated vasculitis, Microscopic polyangiitis, Computed tomography

## Abstract

**Introduction:**

On chest Computed Tomography (CT), pulmonary cryptococcosis and antineutrophil cytoplasmic antibody (ANCA)-associated vasculitis (AAV) can both present as multiple non-cavitating pulmonary nodules. As a result, when such nodules worsen during antifungal therapy in a patient with pulmonary cryptococcosis, it is difficult to determine whether this represents progression of the infection or the development of concomitant AAV, posing a significant diagnostic challenge.

**Case Presentation:**

A 67-year-old man presented with multiple non-cavitating pulmonary nodules and was diagnosed with pulmonary cryptococcosis. He completed a full course of antifungal therapy with clinical and radiologic improvement, and treatment was discontinued. After stopping therapy, he relapsed with the same pattern of multiple non-cavitating pulmonary nodules. Restarting antifungal therapy led to regression of the lesions. Unexpectedly, during antifungal maintenance therapy, he again developed multiple non-cavitating pulmonary nodules. Laboratory testing showed positivity for myeloperoxidase (MPO) and perinuclear anti-neutrophil cytoplasmic antibodies (p-ANCA), supporting a diagnosis of microscopic polyangiitis (MPA), a subtype of AAV. After discontinuation of antifungal therapy and treatment with prednisone plus mycophenolate mofetil, the pulmonary nodules showed marked regression.

**Conclusion:**

Multiple non-cavitating pulmonary nodules can be an atypical presentation of MPA and overlap radiologically with pulmonary cryptococcosis. Accurate differentiation should integrate ANCA serology with careful assessment for involvement of other organ systems.

## 1 INTRODUCTION

Antineutrophil cytoplasmic antibody (ANCA)-associated vasculitis (AAV) comprises a group of disorders characterized by necrotizing inflammation of small vessels, including arterioles, venules, and capillaries. The classic subtypes are Granulomatosis With Polyangiitis (GPA), Microscopic Polyangiitis (MPA), and Eosinophilic Granulomatosis With Polyangiitis (EGPA) [1].

The chest Computed Tomography (CT) features of AAV subtypes are informative and can guide preliminary clinical classification. In GPA, bilateral multiple pulmonary nodules of varying size with ill-defined margins are common. They often lie subpleurally or along vascular bundles and frequently cavitate or necrose, reflecting granulomatous inflammation [2-4]. EGPA typically shows transient, migratory, patchy opacities, sometimes accompanied by small pleural effusions, related to lung tissue inflammation induced by eosinophilic infiltration. [5]. In MPA, diffuse alveolar hemorrhage and interstitial lung disease predominate, presenting as bilateral symmetric or asymmetric ground-glass opacities, with a crazy-paving pattern in some regions [6, 7].

Pulmonary cryptococcosis is a fungal lung infection that occurs in both immunocompetent and immunocompromised individuals. Although its chest CT appearance is diverse, several patterns are characteristic. The most common form is nodular or mass-like, with solitary or multiple nodules typically 0.5–3 cm in diameter, sometimes larger. Margins can be well-defined or ill-defined, and the attenuation is typically homogeneous. A halo sign may appear around some nodules. These lesions are predominantly located in the peripheral lung near the pleura. [8, 9]. A minority of cases show thin-walled, regular cavities. Some patients present with patchy consolidation with irregular contours and indistinct margins, which may involve multiple lobes or segments and contain air bronchograms [8, 9]. A diffuse miliary pattern is less common and appears mainly in severely immunocompromised patients, characterized by evenly distributed 2–5 mm nodules with uniform density and sharp margins throughout both lungs [8, 9].

In clinical practice, the chest CT findings of AAV and pulmonary cryptococcosis each have distinctive features but also overlap substantially. Both can present with pulmonary nodules, and some patients can have pleural effusions, which undoubtedly pose a significant challenge for differential diagnosis. Especially when imaging changes emerge during treatment for pulmonary cryptococcosis, based on imaging alone, it is difficult to distinguish early and rapidly whether the process reflects relapse, antifungal resistance with inadequate disease control, or a newly concurrent AAV.

To clarify the key elements of the differential diagnosis and refine clinical decision-making, particularly when AAV arises during therapy for pulmonary cryptococcosis, we present a detailed analysis of a 67-year-old man. By tracing the dynamic course of his illness during antifungal treatment and the unexpected onset and confirmation of AAV, we aim to offer practical guidance for diagnosing and managing similarly complex cases.

## 2 CASE PRESENTATION

**Fig. (1):**
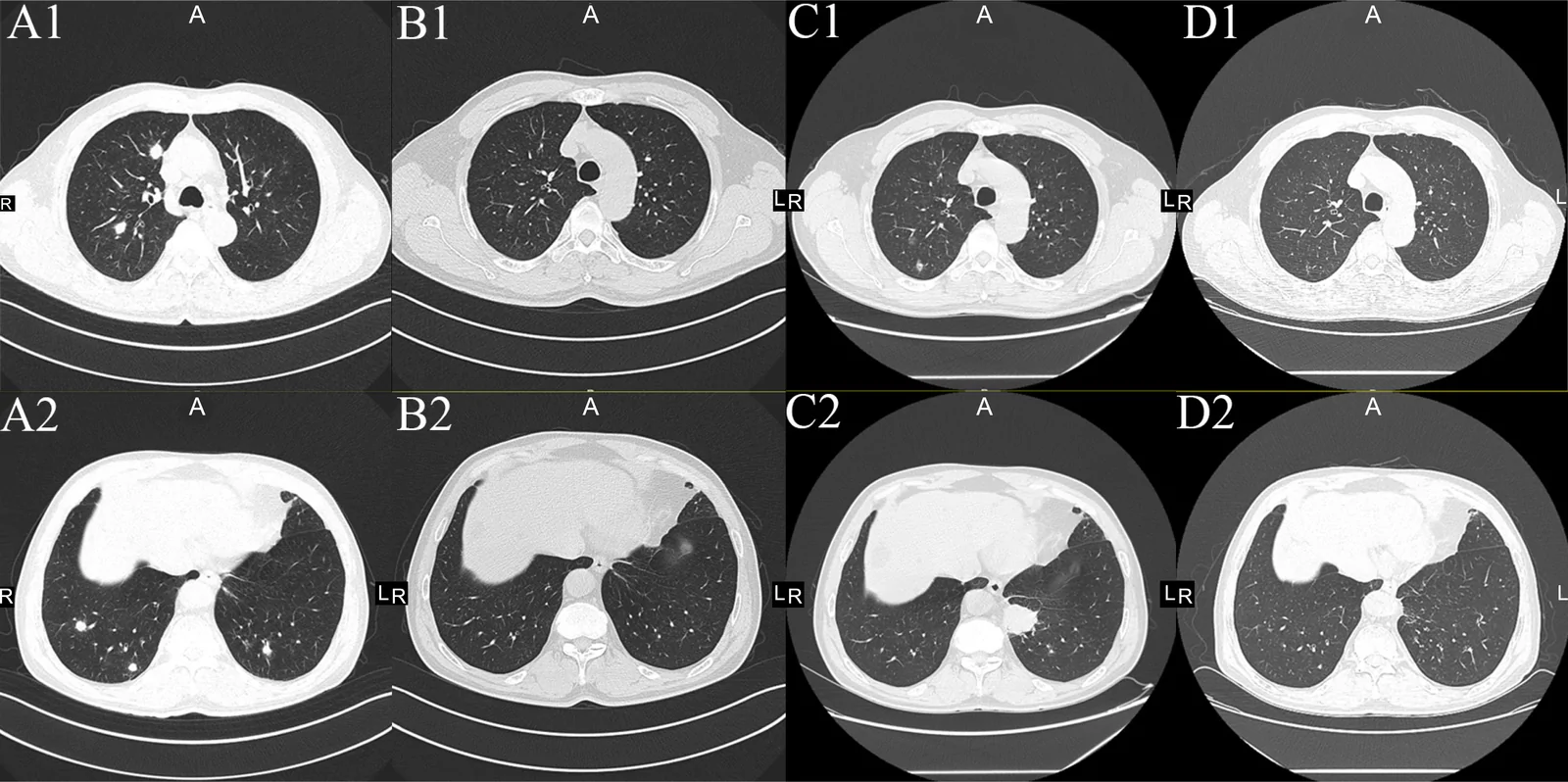
Chest CT. **A1**-**A2**: On 2022-02-07, chest CT showed multiple pulmonary nodules in both lungs, including a larger nodule in the right middle lobe measuring about 18 mm with mildly heterogeneous contrast enhancement. Scattered small, ill-defined nodules were predominant in both upper lobes. **B1**-**B2**: After antifungal therapy, chest CT on 2022-06-24 showed a marked decrease in the number and size of the pulmonary nodules. **C1**-**C2**: Three weeks after discontinuing fluconazole, follow-up chest CT on 2022-08-31 again demonstrated multiple nodules in both lungs, some with ill-defined margins. The largest lesion was paraspinal in the left lower lobe, measuring approximately 31 x 37 mm. Compared with the chest CT on 2022-06-24, the nodules had increased in number and size. **D1**-**D2**: Fluconazole was reintroduced for presumed recurrent cryptococcosis. After eight months of treatment, chest CT on 2023-05-24 showed near-complete resolution of the nodules.

A 67-year-old man had a history of hypertension for over 10 years, treated long-term with amlodipine with good blood pressure control. He reported no smoking or alcohol use and had no notable occupational or environmental exposures. He presented to our hospital with a cough lasting more than two months. Chest CT on 2022-02-07 showed multiple pulmonary nodules in both lungs, including a larger nodule in the right middle lobe measuring about 18 mm with mildly heterogeneous contrast enhancement. Scattered small, ill-defined nodules were predominantly in both upper lobes (Fig. **1 A1**-**A2**). Physical examination was unremarkable. The serum cryptococcal capsular antigen was positive, and a clinical diagnosis of pulmonary cryptococcosis was made. Fluconazole 400 mg orally once daily was initiated. A follow-up chest CT on 2022-06-24 showed a marked decrease in the number and size of the pulmonary nodules (Fig. **1 B1**-**B2**). On 2022-08-09, after six months of fluconazole with complete resolution of cough, treatment was discontinued, and the patient was monitored.

A repeat chest CT on 2022-08-31 revealed multiple nodules in both lungs, some with ill-defined margins. The largest lesion was paraspinal in the left lower lobe, measuring approximately 31 x 37 mm. Compared with the chest CT on 2022-06-24, the nodules had increased in number and size (Fig. **1 C1**-**C2**). Based on the imaging features and prior history, relapse of cryptococcosis was strongly suspected, and fluconazole 400 mg orally once daily was restarted. Serial chest CTs then showed progressive nodule shrinkage, and by 2023-05-24, the multiple nodules had nearly resolved (Fig. **1 D1**-**D2**). Fluconazole was continued thereafter for consolidation therapy.

**Fig. (2):**
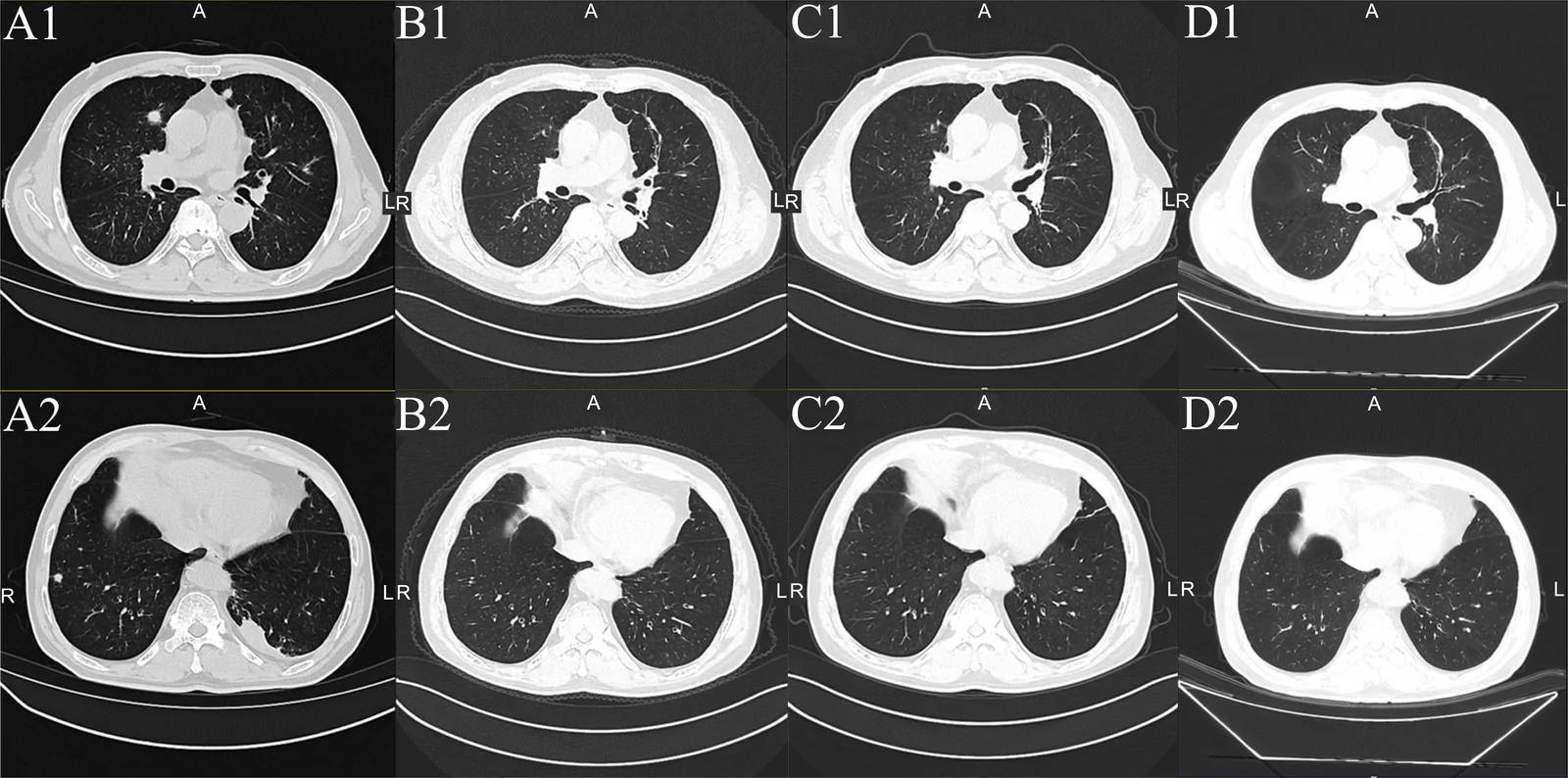
Chest CT. **A1**-**A2**: On 2023-08-23, follow-up chest CT showed progression of multiple bilateral pulmonary nodules compared with 2023-05-24. The largest nodule, in the right upper lobe, measured about 17 mm with ill-defined margins. Numerous small, ill-defined nodules were predominant in both upper lobes, and a small left pleural effusion was present. **B1**-**B2**: After approximately three months of treatment for AAV, chest CT on 2023-12-12 showed marked marked reduction of the bilateral nodules and near-complete resolution of the left pleural effusion. **C1**-**C2**: Follow-up chest CT on 2024-03-19 showed scattered small nodules in both lungs, which were unchanged from 2023-12-12. **D1**–**D2**: Follow-up chest CT on 2025-03-17 showed scattered small nodules in both lungs, which were unchanged from 2024-03-19.

**Fig. (3A-D):**
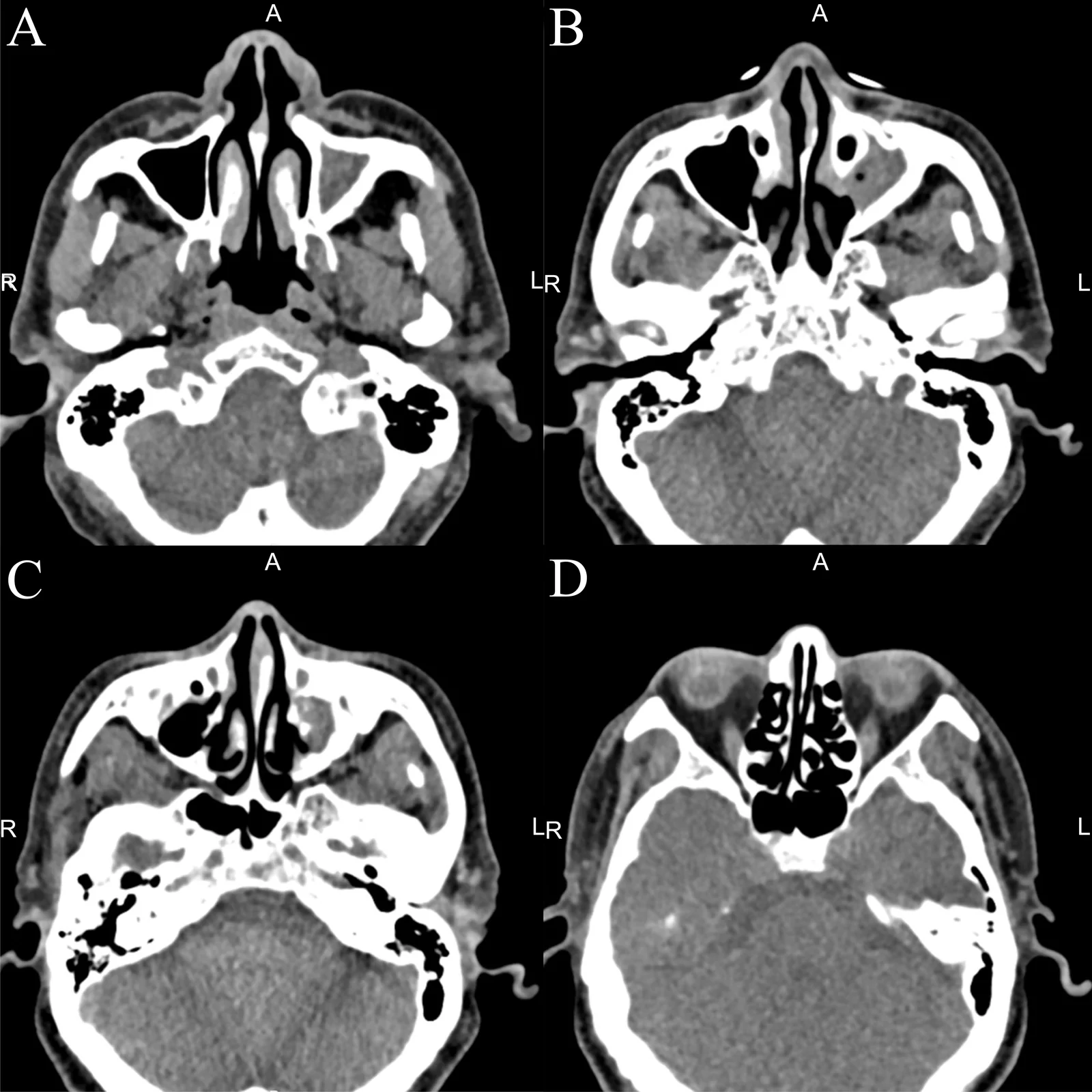
Sinus CT on 2023-09-01 showed high-density material within the left maxillary sinus with smooth bony walls and thickening of the sinus wall. The nasal septum was deviated to the left.

Unexpectedly, while still receiving fluconazole, the patient developed intermittent cough with white sputum in August 2023, without chest tightness, dyspnea, fever, or other symptoms. On 2023-08-23, repeat chest CT showed progression of multiple bilateral pulmonary nodules compared with 2023-05-24. The largest nodule in the right upper lobe measured about 17 mm and had ill-defined margins. Numerous small, ill-defined nodules were predominantly in both upper lobes, and a small left pleural effusion was present (Fig. **2 A1**-**A2**). He was admitted to the hospital and underwent further diagnostic workup. Erythrocyte sedimentation rate was 28 (1–15 mm/h), myeloperoxidase (MPO) 215.0 (0.0–24.0 AU/mL), and perinuclear ANCA (P-ANCA) was positive. Urinalysis showed red blood cells at 19.6 (0.0–18.0/μL), trace hematuria, and no proteinuria. White blood cell count, C-reactive protein, liver and kidney function, electrolytes, troponin I, N-terminal pro–B-type natriuretic peptide, coagulation profile, tumor markers, immunoglobulins (A, G, M), proteinase 3 (PR3), and anti-glomerular basement membrane (anti-GBM) antibodies were within normal limits. The (1,3)-β-D-Glucan test, galactomannan test, serum cryptococcal capsular antigen, cytoplasmic ANCA (C-ANCA), and QuantiFERON-TB Gold test were negative (Table **1**). Serologic tests for hepatitis B, hepatitis C, HIV, and syphilis were negative. Echocardiography showed mild tricuspid and mitral regurgitation with a left ventricular ejection fraction of 67.4%. Sinus CT demonstrated high-density material within the left maxillary sinus with smooth bony walls and thickening of the sinus wall (Fig. **3**).

**Fig. (4):**
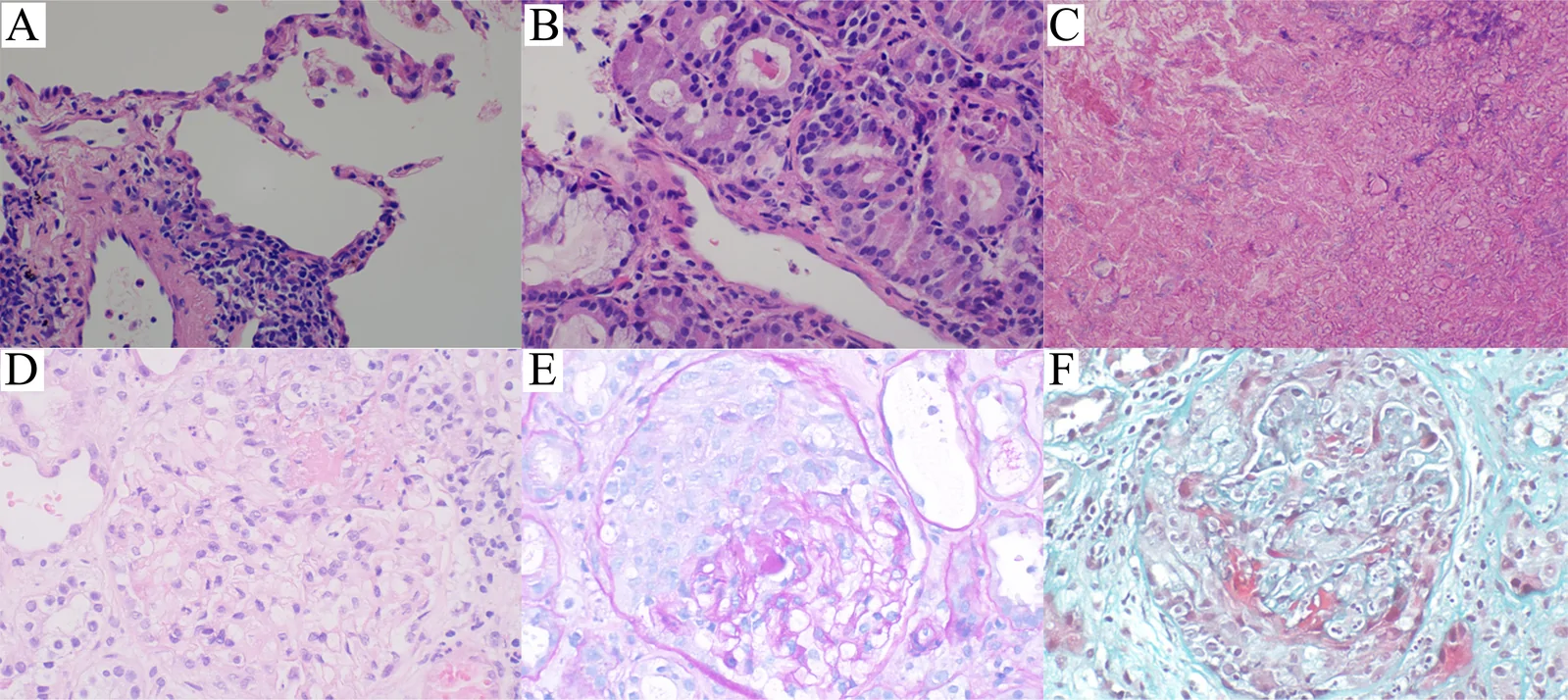
Histopathology. **A** (H&E, 40×), lung biopsy (August 21, 2023): alveolar epithelial hyperplasia with a mixed interstitial infiltrate of lymphocytes, plasma cells, eosinophils, and occasional neutrophils, and focal fibrinous exudate. **B** (H&E, 40×) and C (H&E, 40×), left paranasal sinus specimen (September 8, 2023): interstitial edema with inflammatory cell infiltration and glandular hyperplasia (**B**); fungal colonies (**C**). **D** (H&E, 40×), **E** (Masson, 40×), and **F** (Periodic acid–Schiff, 40×), renal biopsy (March 30, 2025): 32 glomeruli identified, with crescents in 14 (43.8%); glomeruli show focal segmental fibrinoid necrosis, with mild focal segmental proliferation of mesangial and endothelial cells, capillary lumina are poorly patent.

On 2023-08-30, CT-guided percutaneous biopsy of a right upper lobe nodule was performed, yielding three cores. Metagenomic next-generation sequencing (mNGS) on one core detected Klebsiella pneumoniae (three reads). Histopathology on the other two cores showed nonspecific inflammatory changes (Fig. **4A**). Both mNGS and pathology showed no evidence of pulmonary cryptococcosis recurrence, so antifungal therapy with fluconazole capsules was discontinued. Due to the sinus CT suggesting high-density material within the left maxillary sinus, he underwent left endoscopic sinus surgery on 2023-09-05. Postoperative pathology showed chronic inflammation of the left nasal mucosa and a mold ball in the left maxillary sinus, without sinonasal features of ANCA-associated vasculitis (Fig. **4B**,**C**).

No postoperative antifungal therapy was prescribed by the otolaryngology team. Given the diagnosis of MPA and an uneventful postoperative course, treatment with prednisone and mycophenolate mofetil (MMF) was initiated on September 19, 2023. On follow-up after treatment, chest CT on 2023-12-12 showed marked reduction of the bilateral nodules and near-complete resolution of the left pleural effusion compared with 2023-08-23 (Fig. **2 B1-B2**). Prednisone plus MMF was continued, the clinical status remained stable, and renal function tests were normal. Chest CT on 2024-03-19 was essentially unchanged from 2023-12-12 (Fig. **2 C1-C2**). On 2025-03-17, he returned with 10 days of gross hematuria and foamy urine. Tests showed serum creatinine 371 μmol/L, urinalysis with red blood cells 574/μL and hematuria (++), urine microalbumin 438 mg/L (0–19 mg/L), MPO 231.7 AU/mL, P-ANCA positive, and anti-GBM antibodies 200.6 AU/mL. Renal biopsy demonstrated pauci-immune glomerulonephritis, consistent with MPA-related renal involvement (Fig. **4D**-**F**). Chest CT that day showed scattered small nodules, unchanged from 2024-03-19 (Fig. **2 D1–D2**). Although the pulmonary disease remained stable, new MPA-associated kidney injury prompted adjustment of therapy to methylprednisolone plus cyclophosphamide, with continued follow-up.

## 3 DISCUSSION

GPA and MPA arise largely because self-reactive T and B cells lose tolerance to PR3 and MPO within neutrophil cytoplasm, leading to the production of ANCA. These antibodies aberrantly activate neutrophils and trigger inflammation, resulting in vasculitis and tissue injury [10]. In our patient, p-ANCA and MPO-ANCA were positive. The initial manifestations were multiple non-cavitating pulmonary nodules and trace hematuria. Although we diagnosed MPA, diagnostic certainty was limited at first. Treatment with mycophenolate mofetil and prednisone led to a marked reduction in pulmonary nodules and resolution of the pleural effusion, with a favorable clinical response. Convincing evidence for MPA emerged only during follow-up, when the patient developed renal failure; kidney biopsy showed changes consistent with MPA-related renal injury. On this basis, the patient fulfills the laboratory and biopsy domains of the 2022 ACR/EULAR Classification Criteria for MPA [11]. Nasal involvement carries a negative factor in these criteria [11]. Although sinus CT in this patient suggested sinusitis, the lesion was confined to a single frontal sinus, other sinuses were unaffected, and surgical pathology demonstrated fungal sinusitis without features of AAV. We therefore did not assign the negative nasal involvement factor. Taken together, the 2022 ACR/EULAR criteria support classifying this case as MPA.

However, typical chest CT findings in MPA include bilateral, often peripheral, ground-glass opacities, and may also show reticulation, interlobular septal thickening, consolidation, and honeycombing, with a lower-lobe predominance [12, 13]. By contrast, when AAV presents solely with multiple pulmonary nodules, GPA is more common and MPA is uncommon. In our case, during both the period of cryptococcal infection and during active vasculitis, the dominant CT feature remained multiple non-cavitating pulmonary nodules. This atypical pattern in MPA is not unique. Tan *et al*. reported an indolent MPA case that presented with pulmonary nodules as the initial and predominant feature, without clear systemic vasculitic symptoms and with only mildly elevated inflammatory markers [14]. Suzuki *et al*. described a patient initially suspected of having metastatic pulmonary nodules who was ultimately diagnosed with MPA; the nodules gradually shrank and disappeared with effective disease control [15]. Notably, these small nodules closely resemble the pulmonary findings described in our case, namely multiple non-cavitating pulmonary nodules, suggesting that this pattern may represent an atypical manifestation of MPA. In addition, none of these nodules showed obvious cavitation. This phenomenon may be related to MPO-pANCA mediated inflammation, which more readily involves small intrapulmonary arteries and the capillary bed and leads to local aggregation of inflammatory cells and nodule formation. In contrast, inflammation related to PR3-cANCA is more likely to disrupt lung tissue architecture and cause cavitation [16]. This may explain why our MPO-pANCA positive patient had no cavitary lesions. Clinically, a pattern dominated by multiple non-cavitating nodules more often suggests infection (for example, fungal disease or tuberculosis) or malignancy, which understandably led radiologists and clinicians to prioritize recurrent cryptococcosis over an autoimmune flare at the time of relapse.

Pulmonary cryptococcosis overlaps substantially with AAV, especially GPA and MPA with atypical pulmonary findings, which complicates differential diagnosis. Both conditions can present with multiple pulmonary nodules, with or without pleural effusion. Furthermore, the histopathology also overlaps, although there are distinguishing features. Pulmonary cryptococcosis typically shows noncaseating granulomas containing encapsulated yeasts. In contrast, GPA usually shows necrotizing granulomatous inflammation and small-vessel vasculitis [17, 18]. Studies indicate that in lung biopsy specimens from patients with AAV, specific histopathologic features such as classic vasculitis and granulomas are present in only 41.7% of cases [19]. When biopsy material is limited or lesions are atypical, the characteristic features of vasculitis may be less apparent, and inflammatory cell infiltration can be seen in both entities, leading to diagnostic confusion. In our case, the lung needle biopsy showed only inflammatory cell infiltration without definite vascular necrosis or granulomas, so pathology did not immediately indicate AAV. The initial diagnosis of MPA was based on serology (MPO-pANCA positivity) combined with microscopic hematuria. Acute kidney injury then developed during follow-up, and renal biopsy confirmed MPA. These findings highlight that, in patients with multiple non-cavitating pulmonary nodules, particularly when a presumed infection responds poorly to treatment, clinicians should consider autoimmune disease in addition to antimicrobial resistance and should promptly obtain ANCA and related serologic tests. In addition, a comprehensive assessment that incorporates evidence of multisystem involvement is essential.

Among patients with MPO-AAV, more than half of those with interstitial lung disease (ILD) develop renal involvement. Kidney disease usually occurs after the onset of ILD, although in a minority, it appears concurrently or precedes the lung disease [20, 21]. Among these MPO-AAV patients with ILD, 50% had no apparent renal involvement at presentation but were later diagnosed with glomerulonephritis during follow-up, with a median interval of 3.2 years and a maximum of 10 years [20]. Our patient followed a similar course. At the time of MPA diagnosis, there was only mild microscopic hematuria, and renal function remained normal until acute kidney failure occurred 1.5 years later and biopsy confirmed glomerulonephritis. However, because studies focusing on MPAs presenting predominantly with atypical pulmonary nodules are scarce, the temporal relationship between pulmonary nodular lesions and renal involvement in this special subset remains unclear and warrants further investigation.

The causes of AAV are complex and not fully understood. They include primary forms and secondary forms. Drug-induced AAV is one of the secondary causes. Reports have implicated hydralazine, thiamazole, sofosbuvir, minocycline, mirabegron, nintedanib, and antithyroid agents such as propylthiouracil and methyl-mercapto-imidazole derivatives as potential triggers of AAV [22, 23]. However, we found no reports linking fluconazole to AAV, and our patient was not taking other suspect medications, so drug-induced AAV is unlikely. Nevertheless, the potential link between infection and autoimmunity warrants vigilance. In a study of 270 AAV cases and 2,687 controls, Rathmann and colleagues observed a positive association between MPO-ANCA-related vasculitis and respiratory infections [24]. As a chronic fungal infection of the respiratory tract, pulmonary cryptococcosis may promote ANCA-related autoimmunity through persistent antigenic stimulation. Possible mechanisms include the following. Firstly, molecular mimicry or antigenic cross-reactivity between cryptococcal antigens and host targets, which may break immune tolerance. Secondly, chronic infection and inflammation may activate autoreactive T and B cells, enhance presentation of neutrophil-related antigens, and drive autoantibody production, which may induce or amplify ANCA responses [25]. Although a causal relationship between cryptococcal infection and AAV cannot be established in this case, this possibility suggests that clinicians managing patients with chronic infections should monitor dynamic changes in autoimmune markers, especially when infection responds poorly to treatment or new multisystem symptoms emerge.

## CONCLUSION

This case indicates the following: First, multiple non-cavitating pulmonary nodules may represent an atypical pulmonary manifestation of MPA. Second, pulmonary cryptococcosis and AAV can overlap on imaging, such as in the presence of multiple non-cavitating pulmonary nodules, so diagnosis should integrate ANCA serology and evidence of multisystem involvement. A limitation of this case is that we overlooked the possibility of AAV after diagnosing pulmonary cryptococcosis, resulting in the failure to obtain baseline AAV-related antibodies during the initial two diagnostic assessments.

## Figures and Tables

**Table 1:** The results of the hematological tests conducted during the hospitalization of the patient in August 2023.

Blood Test	Results
White Blood Cell Count (3.5-9.5×10^9/L)	7.4
Hemoglobin (130-175g/L)	147
Platelet Count (125-350×10^9/L)	252
C-Reactive Protein (<6.0mg/L)	3.3
Erythrocyte Sedimentation Rate (1-15mm/hr)	28
Alanine Aminotransferase (9-50U/L)	15
Aspartate Aminotransferase (15-40U/L)	20
Albumin (40.0-55.0g/L)	44.8
Serum Creatinine (57-111μmol/L)	67
Troponin I (0-54ng/L)	<2.50
N-terminal pro b-type Natriuretic Peptide (<900pg/mL)	85
Perinuclear Anti-Neutrophil Cytoplasmic Antibodies (P-ANCA)	Positive
Cytoplasmic Anti-Neutrophil Cytoplasmic Antibodies (C-ANCA)	Negative
Myeloperoxidase (MPO)Antibodies (0.0-24.0AU/ml)	215.0
Proteinase 3 Antibodies (PR3) (0.0-24.0AU/ml)	<2.00
Anti-Glomerular Basement Membrane (anti-GBM) Antibodies (0.0-24.0AU/ml)	<2.00
(1,3)-β-D-Glucan Test (<10.00pg/mL)	<5.00
Galactomannan Test (<0.5)	0.153
Serum Cryptococcus Capsular Antigen	Negative
QuantiFERON-TB Gold Test	Non-Reactivity

## Data Availability

The data and supportive information are available within the article.

## LIST OF ABBREVIATIONS

CT: Computed tomography
ANCA: Antineutrophil cytoplasmic antibody
AAV: Antineutrophil cytoplasmic antibody-associated vasculitis
GPA: Granulomatosis with polyangiitis
MPA: Microscopic polyangiitis
EGPA: Eosinophilic granulomatosis with polyangiitis
PR3: Proteinase 3
MPO: Myeloperoxidase
P-ANCA: Perinuclear anti-neutrophil cytoplasmic antibodies
C-ANCA: Cytoplasmic anti-neutrophil cytoplasmic antibodies
GBM: Glomerular basement membrane antibodies
mNGS: Metagenomic next-generation sequencing
ILD: Interstitial lung disease

## STANDARDS OF REPORTING

CARE guidelines were followed.
